# Increase of Long-Term ‘Diabesity’ Risk, Hyperphagia, and Altered Hypothalamic Neuropeptide Expression in Neonatally Overnourished ‘Small-For-Gestational-Age’ (SGA) Rats

**DOI:** 10.1371/journal.pone.0078799

**Published:** 2013-11-12

**Authors:** Karen Schellong, Uta Neumann, Rebecca C. Rancourt, Andreas Plagemann

**Affiliations:** 1 Clinic of Obstetrics, Division of ‘Experimental Obstetrics’, Charité – University Medicine Berlin, Campus Virchow-Klinikum, Berlin, Germany; 2 Medical Center for Women’s and Children’s Health, Department of Pediatrics, Klinikum Ernst von Bergmann, Potsdam, Germany; University of Santiago de Compostela School of Medicine - CIMUS, Spain

## Abstract

**Background:**

Epidemiological data have shown long-term health adversity in low birth weight subjects, especially concerning the metabolic syndrome and ‘diabesity’ risk. Alterations in adult food intake have been suggested to be causally involved. Responsible mechanisms remain unclear.

**Methods and Findings:**

By rearing in normal (NL) *vs.* small litters (SL), small-for-gestational-age (SGA) rats were neonatally exposed to either normal (SGA-in-NL) or over-feeding (SGA-in-SL), and followed up into late adult age as compared to normally reared appropriate-for-gestational-age control rats (AGA-in-NL). SGA-in-SL rats displayed rapid neonatal weight gain within one week after birth, while SGA-in-NL growth caught up only at juvenile age (day 60), as compared to AGA-in-NL controls. In adulthood, an increase in lipids, leptin, insulin, insulin/glucose-ratio (all *p*<0.05), and hyperphagia under normal chow as well as high-energy/high-fat diet, modelling modern ‘westernized’ lifestyle, were observed only in SGA-in-SL as compared to both SGA-in-NL and AGA-in-NL rats (*p*<0.05). Lasercapture microdissection (LMD)-based neuropeptide expression analyses in single neuron pools of the arcuate hypothalamic nucleus (ARC) revealed a significant shift towards down-regulation of the anorexigenic melanocortinergic system (*proopiomelanocortin*, *Pomc*) in SGA-in-SL rats (*p*<0.05). Neuropeptide expression within the orexigenic system (*neuropeptide Y* (*Npy*), *agouti-related-peptide* (*Agrp*) and *galanin* (*Gal*)) was not significantly altered. In essence, the ‘orexigenic index’, proposed here as a neuroendocrine ‘net-indicator’, was increased in SGA-in-SL regarding *Npy/Pomc* expression (*p*<0.01), correlated to food intake (*p*<0.05).

**Conclusion:**

Adult SGA rats developed increased ‘diabesity’ risk only if exposed to neonatal overfeeding. Hypothalamic malprogramming towards decreased anorexigenic activity was involved into the pathophysiology of this neonatally acquired adverse phenotype. Neonatal overfeeding appears to be a critical long-term risk factor in ‘small-for-gestational-age babies’.

## Introduction

Prevalence of obesity, diabetes and accompanying disturbances has increased globally reaching epidemic levels in adults, adolescents and even children [1]–[4]. To prevent the further spread of this epidemic, identifying early risk factors is urgently needed to develop appropriate prevention strategies.

Since the early 1990s, great attention has been given to the association between a low birth weight (LBW) and long-term risk of developing cardiovascular diseases, type 2 diabetes and the metabolic syndrome. The respective ‘small-baby-syndrome’ hypothesis proposes that poor materno-fetal nutrition leads to growth restriction and, consequently, long-lasting programming towards ‘diabesogenic’ alterations [5], [6]. A ‘thrifty phenotype’ acquired *in* *utero* through poor fetal nutrition should enable affected individuals to better adaptation towards reduced food availability in later life [5], [7]. However, when those individuals are exposed to affluent conditions later on, according to the hypothesis this acquired disposition leads to the development of type 2 diabetes, cardiovascular diseases, and the metabolic syndrome.

Many epidemiological studies have confirmed the phenomenological association between low birth weight and later development of symptoms of the metabolic syndrome [8]–[10]. Causal mechanisms, however, of the ‘small-baby-syndrome’ are still unclear. A recent epidemiological study showed that in formerly ‘small babies’, altered dietary habits are linked to the increased risk in later life [11], in line with the ‘thrifty phenotype’ hypothesis. Being small at birth was associated with higher intake of fat at later adult age.

However, a number of studies have reported that individuals born with high birth weight, induced by prenatal overnutrition, are also at increased risk of ‘diabesity’ later on. Meta-analyses demonstrated that both low and high birth weight are associated with increased risk of developing type 2 diabetes and hypertension [12]–[14]. Moreover, long-term risk for overweight, *i.e.*, the most important cardio-metabolic risk factor, has even been shown to be linearly *positively* related to birth weight [15]. The explanation of this developmental paradox remains unclear and the role of prenatal undernutrition and/or low birth weight as *independent* risk factors for the development of ‘diabesity’ and metabolic syndrome later on has to be challenged [16].

A number of animal studies, especially in rodents, were performed to investigate mechanisms of the association between reduced materno-fetal food supply, low birth weight (LBW) and later diseases. Rats with LBW, however, showed rather reduced body weight in the long-term, reduced food intake and normal glucose tolerance [17]–[19]. The fact that findings from animal models do not completely coincide with the observations from epidemiological studies leads to the suggestion that there must be additional factors predisposing to increased ‘diabesity’ risk in later life of ‘small babies’ [16].

Over several years, our group has proposed that neonatal overnutrition after low birth weight might play a decisive role in this scenario [20]. Currently, catch-up growth through ‘rapid’ neonatal weight gain has become a potential mechanism within the ‘small-baby-syndrome’ hypothesis [21]–[23]. To investigate consequences of neonatal overfeeding, *i.e.*, the probably most important cause of rapid neonatal weight gain, the small litter model is a long-established experimental paradigm [24]–[28]. Reduction of litter size in rodents causes increased weight gain during the early postnatal period due to qualitative as well as quantitative overnutrition [29]. Rats raised in small litters display early overweight, increased food intake, impaired glucose tolerance, hyperinsulinemia, hyperleptinemia, and hypertriglyceridemia in later life [25]–[27], [30], [31]. This neonatally acquired phenotype has been linked with permanent dysregulation of neuropeptides critically involved in the central nervous regulation of food intake and body weight [26], [27], [31]–[38].

Interestingly, altered food intake in the long-term has recently also been considered causal for adverse health outcomes in low birth weight humans [11]. Consequently, the question arises whether the increased risk of ‘diabesogenic’ alterations after low birth weight might rather be a consequence of neonatal overnutrition than fetal underfeeding and low birth weight *per se*. Up to now, this has rarely been considered in clinical and experimental studies.

Thus, we established a new, ‘genuine’ rat model of low birth weight to investigate the long-term outcome of ‘small-for-gestational-age’ rats additionally exposed to neonatal overnutrition, as compared to normal neonatal feeding [39]. We examined later food intake both under normal conditions by feeding standard laboratory chow as well as under dietary provocation by exposing the animals to a high-energy/high-fat diet at higher adult age. Long-term metabolic profile was characterized and hypothalamic expression patterns of orexigenic (*Agrp, Gal, Npy*) and anorexigenic (*Pomc*) neuropeptides in single neurons from the arcuate hypothalamic nucleus (ARC) were measured, using lasercapture microdissection (LMD) combined with quantitative real-time PCR to ensure highest possible specificity and sensitivity [40].

## Materials and Methods

### Ethics Statement

All animal procedures were carried out in accordance with the European Communities Council Directive (86/609/EEC) and were approved by the local animal welfare committee (G 0093/02; Lageso Berlin, Germany).

### Animal Model and Study Design

Virgin female Wistar rats (Charles River Laboratories, Sulzfeld, Germany), weighing 200–250 g, were time mated with normal males and delivered spontaneously. Pups were defined as small-for-gestational-age (SGA) if their birth weight was below the lower limit of the 95% confidence interval of the mean birth weight of all pups of the same litter and sex. Pups which had a birth weight within the limits of the 95% confidence interval for litter and sex were assigned as appropriate-for-gestational-age (AGA). The study groups (neonatal overnutrition *vs.* control) were generated by adjusting the litter sizes *per* mother on day 3 of life into litters of only three pups (small litters, SL) or 12 pups (normal litters, NL) through random distribution [24], [32]. SGA rats were raised then in normal (SGA-in-NL) or small litters (SGA-in-SL) until weaning. AGA rats were raised in normal litters, *i.e.*, under normal neonatal feeding conditions, and served as controls (AGA-in-NL).

After weaning (day 21 of life), female rats were housed under standard conditions with 12/12 h inverse light-dark rhythm, controlled temperature (22±2°C) and free access to tap water and standard laboratory chow (commercial control diet for rats; ssniff R/M-H, Soest, Germany, Code V1536–000). Feeding studies were performed from day 470 to 560 (see below). At day 560, animals were sacrificed and tissues and blood were collected.

### Body Weight and Body Composition

Body weight and body length (nose to anus length) and mortality were monitored and recorded throughout life. Relative body weight/body length was evaluated in g/cm. On day 560, body composition was determined by weighing first the carcass mass after stomach and intestine removal. Next, dry mass and fat-free dry mass (FFDM) were determined by drying carcasses to constant weight followed by whole body chloroform extraction in a Soxhlet apparatus. FFDM and body fat were calculated as percentage of carcass mass [41].

### Basal Metabolic Parameters

Blood samples were taken after an overnight fast (16 h) by puncture of the retroorbital plexus under light ether anaesthesia [31] at days 360 and 560 of life to determine basal metabolic parameters. Blood glucose was measured photometrically using the glucoseoxidase-peroxidase (GOD-PAP) method (Dr Lange GmbH, Berlin, Germany). Total plasma cholesterol and plasma triglyceride concentrations were quantified using the cholesterinoxidase-peroxidase (CHOD-PAP) method and the glyceride-3-phosphatoxidase-peroxidase (GPO-PAP) method, respectively (Dr Lange GmbH, Berlin, Germany).

Leptin concentration was quantified using a commercial radioimmunoassay (rat leptin RIA kit, Linco, St. Charles, MO, USA). Recombinant rat leptin (Linco) served as the standard preparation. The intra-assay variation ranged between 2.4–4.6% in a concentration range of 1.6–11.6 µg/l.

For determination of insulin, within one assay a modified commercial radioimmunoassay was performed (Adaltis, Freiburg, Germany). Rat insulin (Novo Nordisk Biolabs, Copenhagen, Denmark) with a biological potency of 21.3 IU/mg was used as standard preparation. The intra-assay coefficient of variation was 4.5–7.4% in a concentration range of 9.2–94.2 mIU/l. The insulin/glucose-ratio was calculated as a measure of peripheral insulin resistance [42].

### Glucose Tolerance Test

Glucose tolerance tests were performed at days 130 and 530 of life. After an overnight period of fasting (16 h), a 20% glucose solution (1.5 g/kg body weight) was injected intraperitoneally. Blood samples were taken at 0, 15, 30, and 90 minutes after glucose loading for determination of blood glucose levels. Using these values, the area under the curve of glucose (AUCG) against time was calculated for each animal [31].

### Food Intake Study

Food intake was studied at older adult age (between days 470 and 560 of life), with individual housing. First, food intake of standard laboratory chow was measured for 30 days (days 470–500 of life). Chow comprised of 9% fat, 33% protein, and 58% carbohydrates with a metabolizable energy content of 3.1 kcal/g (ssniff R/M-H, Soest, Germany, Code V1536-000). For the following 60 days (500–560 day of life), rats were exposed to a palatable high-energy/high-fat (HE/HF) diet containing 34% fat, 23% protein, 43% carbohydrates with a metabolizable energy content of 4.1 kcal/g (specific diet, Code 132006; Altromin, Lage, Germany). This was a modified version of the diet described by Levin et al. and has previously been shown as highly palatable [34], [43]. As both diets have different energy contents caloric intake per day was calculated (kcal/d). Rats were fed *ad libitum* throughout the study period and had free access to tap water. Food intake was recorded daily and body weight measured weekly to the nearest 0.1 g (Sartorius MC 1, Laboratory LC 6200, Sartorius AG, Göttingen, Germany).

### Neuropeptide Expression in the Hypothalamic Arcuate Nucleus (ARC)

#### Lasercapture Microdissection (LMD)

**Figure 1:**
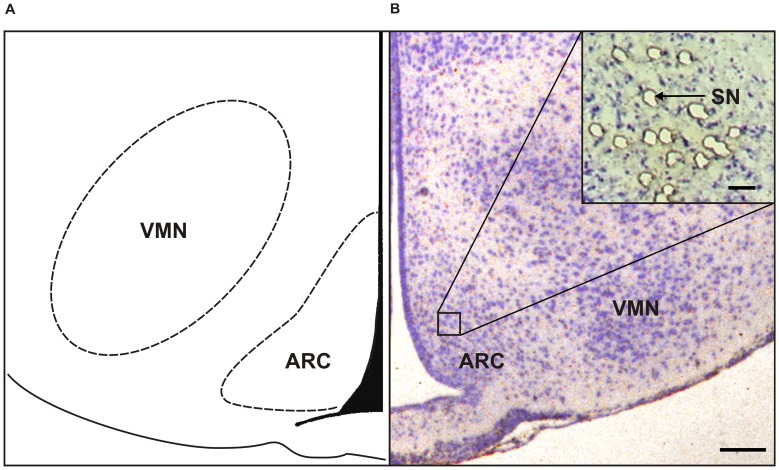
Single neuron preparation using lasercapture microdissection. Schematic illustration (**A**) and Nissl-staining (**B**) of rat hypothalamic nuclei at plane 29 according to Paxinos and Watson [44]; scale bar = 100 µm. Insert shows single neuron preparations from the arcuate nucleus (ARC) using Lasercapture microdissection (LMD); scale bar = 25 µm. VMN = ventromedial hypothalamic nucleus, SN = microdissected single neuron.

Following rapid sacrifice on day 560 of life, brains were immediately isolated, frozen in isopentane and stored at −80°C. For LMD, 10 µm-thick coronary serial sections were cut through the deep-frozen hypothalami, mounted on glass slides (Leica frame slides with 1.4 µm Polyethylene terephthalat (PET)-membrane), dried on air, and finally Nissl-stained with cresyl violet under RNase-free conditions. After staining, slides were kept at −80°C until LMD. Anatomical location of the ARC was verified according to a rat brain atlas [44]. Using the Leica Microsystems AS/LMD® instrument (Leica Microsystems CMS GmbH, Wetzlar, Germany), in total 100 neurons were randomly picked individually from each brain and animal, respectively, pooled from serial sections across the full rostral-caudal extent of the ARC, corresponding to planes 26 to 32 as defined by Paxinos and Watson [44] (Figure 1). In order to ensure neuronal specificity, only neurons with a distinct nucleolus and soma appearance were LMD-prepared and used for subsequent measurements [40]. LMD-captured neuronal cells were additionally verified by microscopical inspection of the tube cap.

#### RNA preparation and quantitative real-time PCR

Total RNA was isolated from LMD-prepared samples and DNase treated using the PureLink RNA Micro Kit (Invitrogen, Carlsbad, USA), according to the manufacturer’s protocol as described previously [40]. RNA was reverse-transcribed into complementary DNA (cDNA) using the Superscript-First-Strand-Synthesis System (Invitrogen), and cDNA was amplified in subsequent real-time PCR.

Duplex real-time PCR was performed in triplicate in an Applied Biosystems 7500 instrument [35], [40]. *Npy, Gal, Agrp,* and *Pomc* mRNA expression were analyzed using commercial intron-spanning TaqMan® gene expression assays from Applied Biosystems (*Npy*: Rn00561681_m1; *Gal*: Rn01501525_m1; *Pomc*: Rn00595020_m1; *Agrp*: Rn01431703_g1; all FAM-labeled), together with an endogenous control assay for the housekeeping gene *Beta actin* (4352340E, VIC-labeled), validated previously [40]. For all amplifications, a standard protocol was used: 1 cycle of 95°C for 10 min, followed by 40 two-step cycles at 95°C for 15 s and 60°C for 1 min [TaqMan Gene Expression Assays Protocol, part number 4333458, Applied Biosystems]. Relative expression of target genes (*vs. Beta actin*) was determined by respective C_t_ values according to the 2^−ΔCt^ method as described elsewhere [40], [45], [46].

### Statistics

Data are expressed as means ± SD unless otherwise indicated. Real-time PCR data are given as arbitrary units. One-way analysis of variance (one-way ANOVA over all groups) followed by Tukey’s HSD *post hoc* analysis was used to analyze group differences (SPSS Software 19.0, Munich, Germany). For analysis of relations between two variables, Spearman’s rank correlation test was performed with GraphPad Prism Version 4.03 (GraphPad Software, Inc., San Diego, California, USA). Statistical significance was set at *p*<0.05.

## Results

### Mortality

Mortality over the entire study period (day 1–560 of life) was increased in neonatally overfed SGA-in-SL rats (12.5%; 2 of 16) as compared to the normal-fed AGA-in-NL rats of the control group (8.5%; 6 of 71), while mortality in neonatally normal-fed SGA-in-NL rats was rather decreased (0%; 0 of 14; differences not statistically significant).

### Body Weight and Body Composition

Mean birth weight of rats born small-for-gestational-age (SGA) was significantly decreased as compared to control rats that had a birth weight appropriate-for-gestational-age (AGA) (*p*<0.001; Table 1). SGA rats exposed to neonatal overnutrition by rearing in small litters (SGA-in-SL) showed rapid neonatal weight gain. From day 7 of life onwards, they did not show any further difference in body weight, as compared to AGA pups raised in normal litters. In contrast, SGA pups raised in normal litters (SGA-in-NL) did not catch up in body weight until day 60 of life. After day 60, no further differences in body weight were observed among the three groups. Additionally, no significant differences in body length and relative body weight were observed in SGA rats raised in normal *vs*. small litters as compared to the rats of the control group until the end of the study (Table 1).

**Table 1:** Body weight, body length and body composition of rats born appropriate-for-gestational-age (AGA) or small-for-gestational-age (SGA).

Variables	AGA-in-NL	SGA-in-NL	SGA-in-SL	*p* (one-way ANOVA)
**Body weight (g)**				
day 1	6.6±0.5 (71)	5.8±0.5^a^ (14)	5.9±0.5^a^ (16)	**<0.001**
day 7	14.1±1.4 (71)	12.4±1.6^a^ (14)	13.8±2.8 (16)	**0.004**
day 14	28.2±2.2 (71)	25.2±4.2^a^ (14)	29.7±6.6^b^ (16)	**0.003**
day 21	46.8±4.3 (71)	40.7±5.8^a^ (14)	47.3±10.0^b^ (16)	**0.001**
day 30	88.7±6.4 (71)	81.6±10.5^a^ (14)	86.2±18.3 (16)	**0.044**
day 60	208±23.4 (71)	208±19.9 (14)	206±33.9 (16)	0.951
day 90	260±22.3 (71)	259±22.0 (14)	255±40.3 (16)	0.763
day 180	310±28.1 (71)	308±29.0 (14)	300±50.5 (16)	0.505
day 360	361±33.1 (70)	363±53.8 (14)	362±65.2 (14)	0.990
day 540	432±56.7 (65)	457±62.3 (14)	460±89.1 (14)	0.177
day 560	444±54.9 (65)	461±56.0 (14)	450±58.6 (14)	0.563
**Body length (cm)**				
day 21	10.1±0.3 (71)	9.9±0.5 (14)	10.3±1.1 (16)	0.108
day 30	13.3±0.4 (71)	12.9±0.6 (14)	13.1±1.3 (16)	0.066
day 60	18.7±0.5 (71)	18.5±0.6 (14)	18.4±1.0 (16)	0.261
day 90	20.2±0.5 (71)	20.1±0.6 (14)	19.9±0.9 (16)	0.170
day 180	21.7±0.6 (71)	21.5±0.8 (14)	21.3±0.7 (16)	0.058
day 360	22.3±0.6 (70)	22.4±0.6 (14)	22.2±0.9 (14)	0.587
day 540	22.2±0.5 (65)	22.2±0.5 (14)	21.9±0.7 (14)	0.117
day 560	22.2±0.5 (65)	22.3±0.5 (14)	21.9±0.7 (14)	0.092
**Body composition (day 560)**				
Relative body weight (g/cm)	20.0±2.2 (65)	20.4±2.0 (14)	20.6±2.3 (14)	0.543
Fat-free dry-mass (%)	19.4±2.0 (64)	19.3±1.8 (14)	19.0±2.6 (14)	0.809
Body fat content (%)	32.5±6.0 (64)	32.0±5.8 (14)	35.5±7.2 (14)	0.223

Values are expressed as means ± SD. Number of animals in parenthesis.

a *p*<0.05 *vs*. AGA-in-NL,

b *p*<0.05 *vs*. SGA-in-NL (by Tukey’s HSD *post hoc* analyses).

Abbreviation: NL, normal litter; SL, small litter.

Analysis of body composition revealed no significant group differences in fat-free dry-mass at day 560, *i.e.*, at the end of the study. However, a trend towards increased body fat content was observed in neonatally overfed SGA-in-SL rats (Table 1).

### Basal Metabolic Parameters

**Figure 2:**
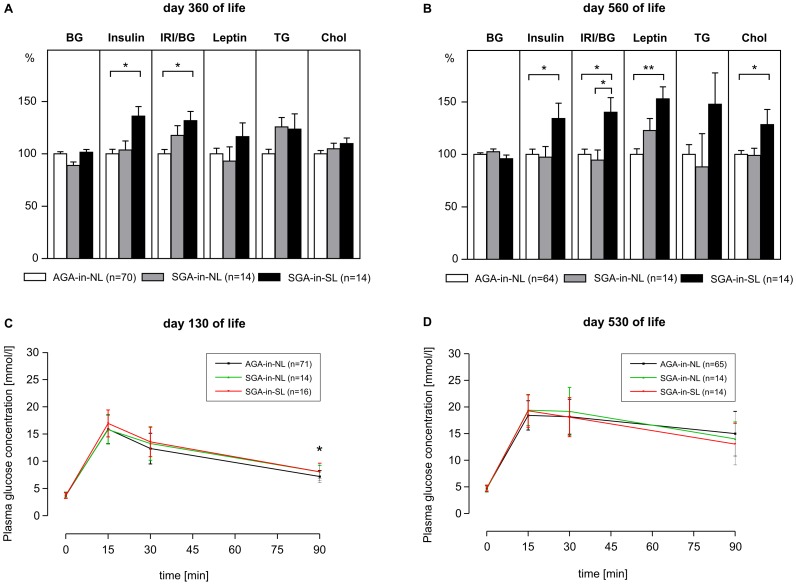
Metabolic parameters in early and later adulthood. Fasting plasma levels of blood glucose (BG), insulin, insulin/glucose-ratio (IRI/BG), leptin, triglycerides (TG), and cholesterol (Chol) on day 360 of life, *i.e.*, before the feeding study (**A**), and day 560 of life, *i.e.*, at the end of HE/HF feeding study (**B**). Data are shown as percentages of AGA-in-NL-levels (means ± SEM). Plasma glucose levels after intraperitoneal glucose loading on day 130 of life, *i.e.*, before feeding study (**C**), and day 530 of life, *i.e.*, during high-energy/high-fat (HE/HF) feeding study (**D**) in rats born small-for-gestational-age, raised in normal litters (SGA-in-NL) or small litters (SGA-in-SL), as compared to rats with normal birth weight raised in normal litters (AGA-in-NL). Data are means ± SD. *p<0.05, **p<0.01 (one-way ANOVA followed by Tukey’s HSD *post hoc* analysis).

**Figure 3:**
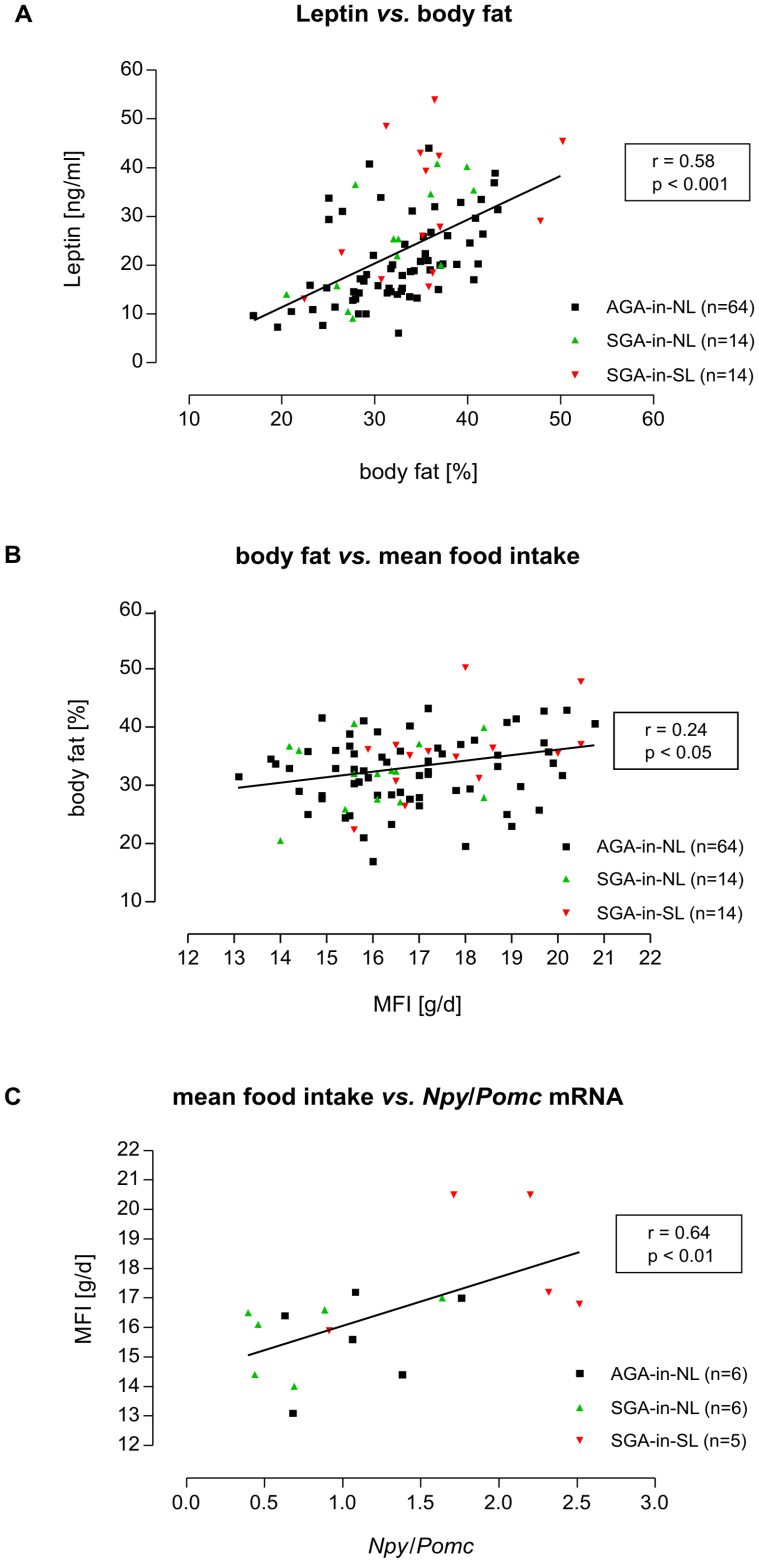
Correlation analyses. Relation between plasma levels of leptin and percentage of body fat at day 560 of life (**A**). Percentage of body fat presented as a function of overall mean food intake (chow+HE/HF) (**B**). Relation between overall mean food intake (chow+HE/HF diet) and mRNA expression of *Npy per* unit *Pomc* (*Npy*/*Pomc*) in the arcuate hypothalamic nucleus at day 560 of life (**C**). Group-specific plots are illustrated (▪: AGA-in-NL; ▴: SGA-in-NL; ▾: SGA-in-SL). Inserts show overall correlation coefficients and significances derived from Spearman’s rank correlation tests.

While basal, *i.e.*, fasting blood glucose levels did not differ significantly between groups over the entire observational period, plasma insulin concentrations were significantly increased in neonatally overnourished SGA-in-SL rats on days 360 of life, *i.e.*, before the feeding study, and 560 of life, *i.e.*, at the end of the HE/HF feeding study, as compared to AGA-in-NL control rats (day 360: AGA-in-NL: 29.3±10.6 mIU/l *vs.* SGA-in-NL: 30.5±9.7 mIU/l *vs.* SGA-in-SL: 39.9±13.7 mIU/l; day 560: AGA-in-NL: 84.8±34.2 mIU/l *vs.* SGA-in-NL: 82.7±30.4 mIU/l *vs.* SGA-in-SL: 113.7±62.3 mIU/l; both *p*<0.05; Figure 2A and 2B). Basal hyperinsulinemia was associated with a significantly increased insulin/glucose-ratio in SGA-in-SL rats (day 360: AGA-in-NL: 6.4±2.3 *vs.* SGA-in-NL: 7.6±2.6 *vs.* SGA-in-SL: 8.5±2.8; day 560: AGA-in-NL: 15.4±6.3 *vs.* SGA-in-NL: 14.6±5.3 *vs.* SGA-in-SL: 21.6±11.3; both *p*<0.05; Figure 2A and 2B). This was accompanied by hyperleptinemia (*p*<0.01), significantly correlated with body fat (Figure 3A), significantly increased cholesterol levels (*p*<0.05) and slightly increased levels of triglycerides at day 560 of life in SGA-in-SL rats. In contrast, at no time point were significant differences in metabolic profile found in SGA rats raised in normal litters (SGA-in-NL) as compared to controls (AGA-in-NL).

### Glucose Tolerance Test

After intraperitoneal glucose loading at younger adult age (day 130 of life), neonatally overnourished SGA-in-SL rats showed significantly increased blood glucose levels at 90 minutes (*p*<0.05; Figure 2C), while their AUCG was not significantly increased (AGA-in-NL: 15.8±2.7 mmol/l/h *vs.* SGA-in-NL: 16.8±2.8 mmol/l/h *vs.* SGA-in-SL: 17.2±2.4 mmol/l/h; *p* = 0.097). Glucose tolerance test at later adult age (day 530 of life) revealed no significant differences between groups (Figure 2D).

### Food Intake

**Figure 4:**
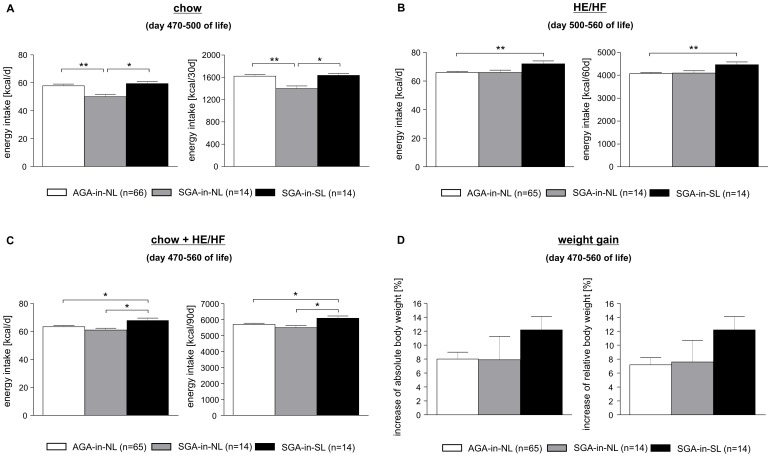
Food intake study in later adult age. *Ad libitum* energy intake of standard laboratory chow for 30 days (day 470–500 of life) (**A**), followed by providing a high-energy/high-fat (HE/HF) diet for 60 days (day 500–560 of life) (**B**). (**C**) shows overall caloric intake of chow and HE/HF diet throughout food intake study (day 470–560 of life). Absolute and relative body weight changes during the food intake study (day 470–560 of life) (**D**)**.** Data are means ± SEM, shown as percentages of AGA-in-NL-levels. *p<0.05, **p<0.01 (one-way ANOVA followed by Tukey’s HSD *post hoc* analysis).

Energy intake in adulthood of standard laboratory chow (days 470–500 of life) was significantly decreased in neonatally normal-fed SGA rats (SGA-in-NL) as compared to both SGA-in-SL and AGA-in-NL rats (AGA-in-NL: 57.9±9.0 kcal/d *vs.* SGA-in-NL: 50.1±6.1 kcal/d *vs.* SGA-in-SL: 59.4±5.3 kcal/d; *p*<0.05 and p<0.01, respectively; Figure 4A). However, SGA-in-NL rats consumed similar calories of high-energy/high-fat diet (days 500–560 of life) as compared to AGA-in-NL animals, while neonatally overfed SGA rats showed significantly increased energy intake of HE/HF diet (AGA-in-NL: 65.9±6.8 kcal/d *vs.* SGA-in-NL: 65.9±6.2 kcal/d *vs.* SGA-in-SL: 72.0±7.5 kcal/d; *p*<0.01; Figure 4B). Energy intake over the whole observational period (day 470–560 of life) was significantly increased in SGA-in-SL rats as compared to SGA-in-NL rats (AGA-in-NL: 63.5±6.7 kcal/d *vs.* SGA-in-NL: 61.1±5.1 kcal/d *vs.* SGA-in-SL: 68.1±5.9 kcal/d; *p*<0.05; Figure 4C), and positively correlated to body fat over all groups (Figure 3B). Accordingly, leptin was positively correlated to body fat not just in SGA-in-SL (r = 0.42) but in all groups (AGA-in-NL: r = 0.58; SGA-in-NL: r = 0.68; Figure 3A).

### Neuropeptide Expression in the Hypothalamic Arcuate Nucleus (ARC)

**Figure 5:**
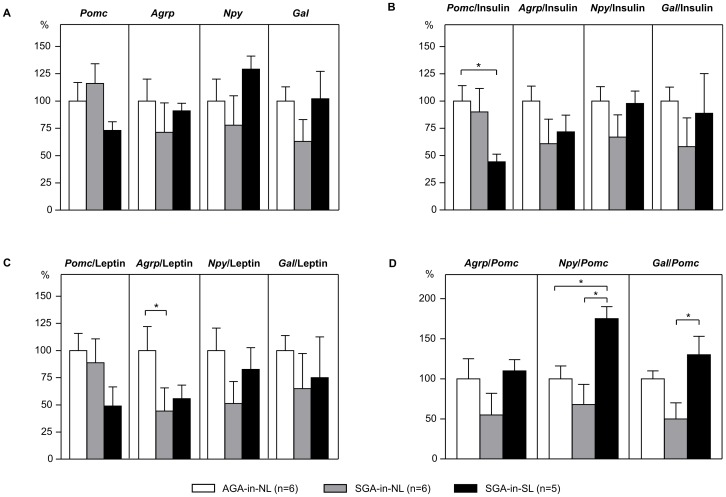
Neuropeptide mRNA expression in neuron pools of the hypothalamic ARC nucleus at day 560 of life. Relative gene expression of *proopiomelanocortin* (*Pomc*), *agouti-related peptide* (*Agrp*), *neuropeptide Y* (*Npy*), and *galanin* (*Gal*), all normalized to *Beta actin* (**A**). Additionally, expression levels at the end of the feeding study (day 560 of life) are shown *per* unit plasma insulin (**B**) and plasma leptin (**C**), respectively, in AGA and SGA rats raised in normal (NL) or small litters (SL). Illustration of ‘orexigenic indices’, calculated as quotients of orexigenic (*Agrp*, *Npy*, *Gal*) *per* anorexigenic (*Pomc*) mRNA expression (**D**). Data are means ± SEM, shown as percentages of AGA-in-NL-levels. *p<0.05 (one-way ANOVA followed by Tukey’s HSD *post hoc* analysis).

Analyses of mRNA expression of neuropeptides in single neuron pools (n = 100 neurons *per* animal) from the ARC at the end of the experiment on day 560 of life revealed no significant differences between groups (Figure 5A). Increased overall food intake in neonatally overnourished SGA-in-SL rats, however, was accompanied by a non-significant tendency towards decreased levels of anorexigenic *Pomc* and increased levels of orexigenic *Npy* expression as compared to AGA-in-NL control rats (*Pomc*: AGA-in-NL: 0.86±0.15 *vs.* SGA-in-NL: 1.00±0.17 *vs.* SGA-in-SL: 0.63±0.05, *p* = 0.213; *Npy*: AGA-in-NL: 0.9±0.2 *vs.* SGA-in-NL: 0.7±0.2 *vs.* SGA-in-SL: 1.2±0.1, *p* = 0.206; Figure 5A). Expression of *Agrp* and *Gal* was unchanged. Neonatally normal-fed SGA-in-NL rats exhibited decreased expression (not statistically significant) of orexigenic *Npy, Agrp* and *Gal* (*Agrp*: AGA-in-NL: 0.15±0.03 *vs.* SGA-in-NL: 0.11±0.03 *vs.* SGA-in-SL: 0.14±0.01, *p* = 0.525; *Gal*: AGA-in-NL: 0.08±0.01 *vs.* SGA-in-NL: 0.05±0.01 *vs.* SGA-in-SL: 0.08±0.02, *p* = 0.306; Figure 5A), corresponding to their overall tendency towards reduced food intake under chow diet (Figure 4).

Because of the well-known dependency of neuropeptide expression (*Pomc*, *Agrp*, *Npy*, *Gal*) on their regulating hormones leptin and insulin [47]–[50], we additionally calculated the quotient of neuropeptide expression *per* unit of leptin and insulin, respectively, as described elsewhere [32]. In SGA-in-SL rats, *Pomc* expression *per* corresponding insulin was clearly decreased as compared to AGA-in-NL rats (AGA-in-NL: 13.6±1.9 *vs.* SGA-in-NL: 12.2±2.6 *vs.* SGA-in-SL: 6.0±0.4; *p*<0.05), whereas the above mentioned non-significant increase in *Npy* expression was no longer present (AGA-in-NL: 13.8±1.8 *vs.* SGA-in-NL: 9.2±1.9 *vs.* SGA-in-SL: 13.5±1.5; *p* = 0.158; Figure 5B). In contrast, SGA-in-NL rats showed a marked decrease in *Agrp* expression *per* corresponding leptin as compared to controls (AGA-in-NL: 10.9±2.4 *vs.* SGA-in-NL: 4.8±1.0 *vs.* SGA-in-SL: 6.1±0.8; *p*<0.05; Figure 5C). In general, the trend towards decreased expression of orexigenic *Npy*, *Agrp*, and *Gal* in SGA-in-NL rats remained even when referring to insulin and leptin, respectively (Figure 5B and 5C). Expression of anorexigenic *Pomc* was unchanged in SGA-in-NL rats, even when referred to insulin and leptin, respectively (Figure 5B and 5C).

Finally, quotients of orexigenic (*Agrp*, *Npy*, *Gal*) *per* anorexigenic (*Pomc*) mRNA expression were calculated to get a proxy of the ‘net-balance’ here [Figure 5D]. *Gal*/*Pomc* was increased significantly in SGA-in-SL as compared to SGA-in-NL rats. Moreover, *Npy*/*Pomc* was found to be nearly doubled in SGA-in-SL as compared to both AGA controls as well as SGA-in-NL rats, and found to be positively correlated to food intake over all groups (Figure 3C).

## Discussion

Low birth weight (IUGR, SGA) has been shown to be related to increased long-term ‘diabesity’ risk though reasons remain unclear. Acquired alterations of food intake have been suggested as a possible mechanism. In most of epidemiological-clinical studies, *in utero* causes of low birth weight and, especially, the potential impact of neonatal nutrition and resulting early growth pattern for the long-term outcome have not been adequately considered. We investigated the impact of neonatal overfeeding for the long-term ‘diabesity’ risk, food intake, and related neuropeptidergic regulatory parameters of body weight control in ‘small-for-gestational-age’ rats, introducing a novel ‘genuine’ rodent LBW-model set according to clinical definitions of SGA (birth weight below the lower limit of the 95% confidence interval of the mean birth weight of same litter and sex).

In our study, all animals with a reduced birth weight (SGA) showed catch-up growth, irrespective of whether they were neonatally normal-fed or overfed. Adult age SGA rats did not differ in total body weight and body length when compared to AGA control rats. This is consistent with epidemiological observations which described that 80–90% of SGA newborns show full catch-up growth within the first two years of life [51], [52]. However, while neonatally normal-fed SGA pups only caught up in body weight at day 60 of life, neonatal overnutrition of SGA pups resulted in ‘rapid’ neonatal weight gain within some days. From the first week of life onwards, these SGA-in-SL rats did not further differ from AGA controls.

Epidemiological-clinical studies have indicated that ‘rapid’ neonatal weight gain appears to be a risk factor for the development of obesity, increased body fat, insulin resistance, impaired glucose tolerance and cardiovascular diseases in the long-term [8], [20], [53]–[58], even independent of birth weight [23], [59]. In a large population-based study, Stettler and colleagues examined the extent to which the development of overweight depends on birth weight and/or weight gain during the first 4 months of life. They observed that increased weight gain from birth until the age of 4 months is associated with later increased overweight risk [59]. This association has been completely confirmed in prenatally ‘overfed’ (hyperglycemia-exposed) offspring of diabetic mothers [23]. By these studies, rapid neonatal weight gain has been shown to be an *independent* risk factor in general as well as in particular at risk populations [23], [59].

According to the ‘small-baby-syndrome’ hypothesis [6], children with a low birth weight are *per se* at increased risk to develop metabolic disturbances in later life, *e.g*., impaired glucose tolerance and dyslipidemia, resulting from prenatal undernutrition. Neonatally normal-fed SGA rats in the present study, however, did not show increased risk throughout later life. In contrast, during glucose tolerance test at day 130 of life, neonatally overnourished SGA-in-SL rats showed significantly increased blood glucose levels at 90 minutes. This tendency towards altered metabolism became further accentuated in older animals, when neonatally overfed SGA-in-SL rats developed hyperinsulinemia and an increased insulin/glucose ratio under basal conditions (days 360 and 560 of life), indicating insulin resistance [42]. Consequently, metabolic alterations observed in SGA rats cannot be attributed to decreased birth weight *per se*, but suggest rather early postnatal overnutrition as the critical risk factor here. This appears to be in line with findings of a clinical study in which the influence of prematurity on later occurrence of insulin resistance has been studied [9]. Hofman et al. observed that children born with low birth weight had reduced insulin sensitivity later on, irrespective of their gestational age (preterm AGA or term SGA). They found that the risk among preterm AGA-children was similar to the risk of term SGA-children. This observation gives rise to reasonable doubts concerning the role of diminished prenatal food supply as an *independent* risk factor in the pathogenesis of ‘diabesity’ in the ‘small-baby-syndrome’ [60]. It can be assumed that rather postnatal influences, especially neonatal overnutrition leading to rapid neonatal weight gain, are of critical importance for the long-term ‘diabesogenic’ outcome of ‘SGA babies’. Accordingly, the association between rapid neonatal weight gain and later metabolic disorders was examined in a prospective cohort study. Fabricius-Bjerre et al. observed that accelerated growth during the first three months of life leads to disturbances of glucose metabolism later in life [8]. High infant weight gain was positively related with high insulin levels as well as high HOMA-IR later on (homeostasis model assessment of insulin resistance).

In addition to the above mentioned diabetic alterations at older adult age in neonatally overfed SGA rats, these animals also showed an altered fat metabolism. SGA-in-SL rats displayed increased levels of cholesterol and triglycerides and clearly increased leptin levels, while SGA-in-NL rats did not show any adipogenic alterations. Hyperleptinemia in neonatally overnourished rats was accompanied by a tendency towards increased body fat content which was, however, not associated with significantly increased total body weight, finally indicating increased ‘adiposity’ in these animals. Thus, elevated leptin levels in SGA-in-SL appear to reflect increased body fat, not necessarily increased total body weight, which is underlined by a positive correlation of body fat with plasma leptin levels (Figure 3A). Findings from human studies support this relationship. For instance, Ibánez et al. have demonstrated that children who were born SGA at term have abdominal fat mass at 4 years closely related to the rate of catch-up weight gain within the first 2 years of life [57]. It is well known from a number of clinical studies that increased fat mass decisively contributes to the development of insulin resistance [61]–[63], accompanied with increased plasma insulin and triglyceride levels [63], as observed here experimentally.

In the context of the pathogenesis of the ‘small-baby-syndrome’, a recently published cohort study considered altered dietary habits in later life to be causal for adverse health outcomes. Being small at birth was associated with higher intake of fat at later adult age [11]. This confirms findings of another longitudinal study in which an inverse relation between birth weight and fat intake in 43-month-old children was described [64]. Interestingly, these epidemiological findings seem to be confirmed by data from our animal experiment. However, the clinical studies did not consider neonatal nutrition and growth pattern. In our experimental study, only SGA rats which were neonatally overfed showed increased food intake later on, especially under high-energy/high-fat diet. Worthy to note, milk of SL dams has been characterized to be altered in terms of a high-energy/high-fat quality as compared to milk of dams nourishing litters of normal size [29]. Therefore, our observations might even indicate an early food preference conditioning of SGA-in-SL rats through their dams’ milk composition towards a HE/HF diet preference, persisting throughout later life. Trend towards elevated body fat content observed in later life of SGA-in-SL rats was possibly caused by hyperphagia and high-energy/high-fat food preference, confirmed by positive correlation between body fat and food intake (Figure 3B). In contrast, neonatally normal-fed SGA-in-NL rats did not show hyperphagia, neither under chow nor under high-energy/high-fat diet. However, a trend towards ‘relative preference’ for HE/HF diet *vs*. chow was observed within the SGA-in-NL group, although neither indicating hyperphagia nor HE/HF preference as compared to AGA or SGA-in-SL rats (Figure 4).

In the presented study, neonatally normal- and overfed SGA rats did not differ significantly with respect to mortality. However, in SGA-in-NL rats mortality was trending to be even lower as compared to AGA controls while, in contrast, SGA-in-SL rats showed a tendency towards increased mortality. This appears to be in line with findings by Ozanne and Hales [65]. While in their studies pre- and neonatal underfeeding gave rise to increased longevity, decreased life span of male mice that underwent fetal growth restriction and thereafter experienced rapid catch-up growth has been observed [65]. Note, in the mentioned experiment [65] reduced birth weight was induced by maternal protein-restriction during pregnancy. Rodent models of maternal malnutrition during pregnancy and lactation are among the most frequently used animal models to investigate mechanisms of perinatal programming according to the ‘small-baby-syndrome’ hypothesis. However, offspring whose dams were fed a low protein diet during pregnancy did not always exhibit the full spectrum of metabolic and cardiovascular alterations as observed in epidemiological and clinical studies. Moreover, experiments mostly were carried out exclusively in males and/or offspring were not examined into older adult age [18], [19], [65]. Therefore, in our study we focused on females and later adult aged animals to properly examine the long-term outcomes.

Food intake and body weight are decisively regulated by orexigenic and anorexigenic neuropeptides expressed in the hypothalamic arcuate nucleus (ARC) [66]. The hypothalamic expression of these neuropeptides is mainly regulated by the circulating satiety signals leptin and insulin [50]. Lasercapture microdissection of single neurons combined with quantitative PCR has been proven to be the most powerful technique allowing a highly precise and complex analysis of gene expression patterns in discrete neuronal cell populations [40]. Therefore, we applied this method here to perform gene expression analyses. Measurements at older adult age revealed a trend towards down-regulation of orexigenic *Agrp* and *Npy* in neonatally normal-fed SGA-in-NL rats whereas expression of the anorexigenic *Pomc* was slightly increased. Expression of orexigenic *Gal* was rather decreased in SGA-in-NL. This is of particular interest since *Gal* is known to particularly stimulate fat ingestion [48], [67]. Altogether, results show a long-term *decreased* activity of the orexigenic system in neonatally normal-fed SGA-in-NL rats, which is strengthened by consideration of circulating leptin and insulin levels (Figures 5B and 5C) and might be causal for the significantly decreased food intake as compared to controls, especially under chow diet.

In contrast, hypothalamic expression of *Agrp* and *Gal* was unchanged and *Npy* even slightly increased in neonatally overfed SGA-in-SL rats as compared to AGA controls, despite their marked basal hyperleptinemia and hyperinsulinemia. Corresponding expression of the anorexigenic *Pomc* was even decreased, also when referred to the increased levels of the satiety signals insulin and leptin. This gene expression pattern strongly indicates a neonatally acquired neuropeptidergic malprogramming, especially of the anorexigenic *Pomc*-system, due to neonatal overfeeding in SGA rats (SGA-in-SL).

Finally, since food intake is regulated by both orexigenic and anorexigenic neuropeptides, we additionally introduced here an integrative neuropeptidergic ‘net-indicator’. Dividing the expression levels of *Npy*, the most potent orexigenic neuropeptide [66], by the expression levels of *Pomc*, the most important anorexigenic neuropeptide [66], may provide an orientating proxy (‘orexigenic index’) for better estimation of neuropeptidergic appetite *vs*. satiety activity and regulation in a given situation. In neonatally overfed SGA-in-SL rats, reduced expression of anorexigenic *Pomc* and unchanged expression of orexigenic *Npy* resulted in an increased ‘orexigenic index’ (*Npy/Pomc*), corresponding with hyperphagia and supported by a positive correlation with overall food intake. Similar was observed for the *Gal*/*Pomc* index (Figure 5D). In contrast, in neonatally normal-fed SGA-in-NL no respective alterations were observed as compared to AGA controls (Figure 5D).

In summary, neonatally normal-fed SGA rats (SGA-in-NL) growth caught up only at late juvenile age and did not develop ‘diabesity’ and hyperphagia later on, neither under normal chow diet nor under high-energy/high-fat dietary provocation representing a ‘westernized’ lifestyle. Their long-term hypothalamically driven orexigenic activity was rather decreased than increased. In contrast, neonatally overfed SGA-in-SL rats displayed rapid neonatal weight gain and catch-up growth within the first week of postnatal life. In the long-term, these SGA rats displayed significantly increased ‘diabesity’ risk as compared to normal rats. Hyperphagia, particularly pronounced under high-energy/high-fat dietary provocation, was accompanied with hyperleptinemia, hyperinsulinemia, increased insulin-glucose-ratio, and correlated with body fat. This was accompanied with and correlated to reduced expression of the anorexigenic hypothalamic ARC-*Pomc*, and respective increase of the ‘orexigenic index’ (*Npy*/*Pomc*, *Gal/Pomc*), even under consideration of the circulating regulators insulin and leptin. Altogether, this indicates a neonatally acquired hypothalamic resistance of the anorexigenic system towards peripheral satiety signals (insulin, leptin) in neonatally overfed SGA-in-SL rats.

In conclusion, the early neonatal period appears to be at least as critical as prenatal life for long-term programming of ‘diabesity’ risk and altered food intake in SGA rats, as we previously suggested and proposed [16], [20], [31], [33], [39]. Neonatal overfeeding may predispose *via* hypothalamic malprogramming to hyperphagia and accompanying/subsequent disorders in terms of the metabolic syndrome in ‘small-for-gestational-age’ subjects. This should be considered in future experimental as well as clinical approaches to unravel mechanisms underlying the ‘small-baby-syndrome’.
